# Primary Pancreatic Lymphoma in the Tail: A Rare Anatomic Presentation

**DOI:** 10.7759/cureus.31709

**Published:** 2022-11-20

**Authors:** Pooja Kothari, Adam Haines, Meera P Bhardwaj, Viraj V Patel, Sammy Ho

**Affiliations:** 1 Internal Medicine, Montefiore Medical Center, New York City, USA; 2 Gastroenterology, Montefiore Medical Center, New York City, USA

**Keywords:** cancer diagnosis, non-hodgkin lymphoma nhl, endoscopic ultrasound (eus), pancreas, primary pancreatic lymphoma

## Abstract

Non-Hodgkin’s lymphoma is a common type of cancer, whose most common site of extranodal involvement is the gastrointestinal tract. However, primary presentation in the pancreas remains uncommon. Among cases with pancreatic involvement, the disease is often found in the head and rarely in the tail. Here, we present a case of a 56-year-old male patient with acute epigastric pain, early satiety, and abdominal distention. CT imaging showed a mass of the pancreatic tail with surrounding lymphadenopathy, concerning lymphoma. Endoscopic ultrasound-guided fine needle aspiration (EUS-guided FNA) diagnosed mature B-cell lymphoma, meeting novel diagnostic criteria for the rare diagnosis of primary pancreatic lymphoma (PPL).

## Introduction

Primary pancreatic lymphoma (PPL) is a rare clinical disease, whereas non-Hodgkin lymphoma is the most common type [1]. PPL comprises less than 0.5% of all pancreatic cancers and less than 2% of extranodal lymphomas [2]. Generally, PPL is found in the pancreatic head, which has a large amount of lymphoid tissue, though it can be found anywhere along the gland [3-5]. There have been few case reports discussing anatomical variation [6,7], but only one case report has been published so far describing PPL localized to the tail [8]. This is a case report that discusses the finding of PPL solely in the pancreatic tail.

## Case presentation

A 56-year-old male with a history of hypertension, hyperlipidemia, and prior cerebral vascular accident (CVA) initially presented to the hospital with one week of severe epigastric abdominal pain. The patient's pain was nearly constant and associated with a few days of loose yellow-brown diarrhea one to two weeks prior to admission. His episodes of diarrhea were followed by two black-appearing bowel movements one week prior to admission. On further review of systems, he endorsed three months of preceding abdominal distension and two weeks of early satiety. He did not have fever, chills, unintentional weight changes, night sweats, nausea, vomiting, diarrhea, or constipation.

Upon presentation to the hospital, his vital signs were within normal limits, and his exam was notable for abdominal distension without tenderness to palpation. Lab investigations were notable for normocytic anemia with a hemoglobin of 13.7 g/dl at presentation. The rest of his laboratory studies including white blood cell count, platelets, liver function tests, and lipase were within normal limits.

**Figure 1:**
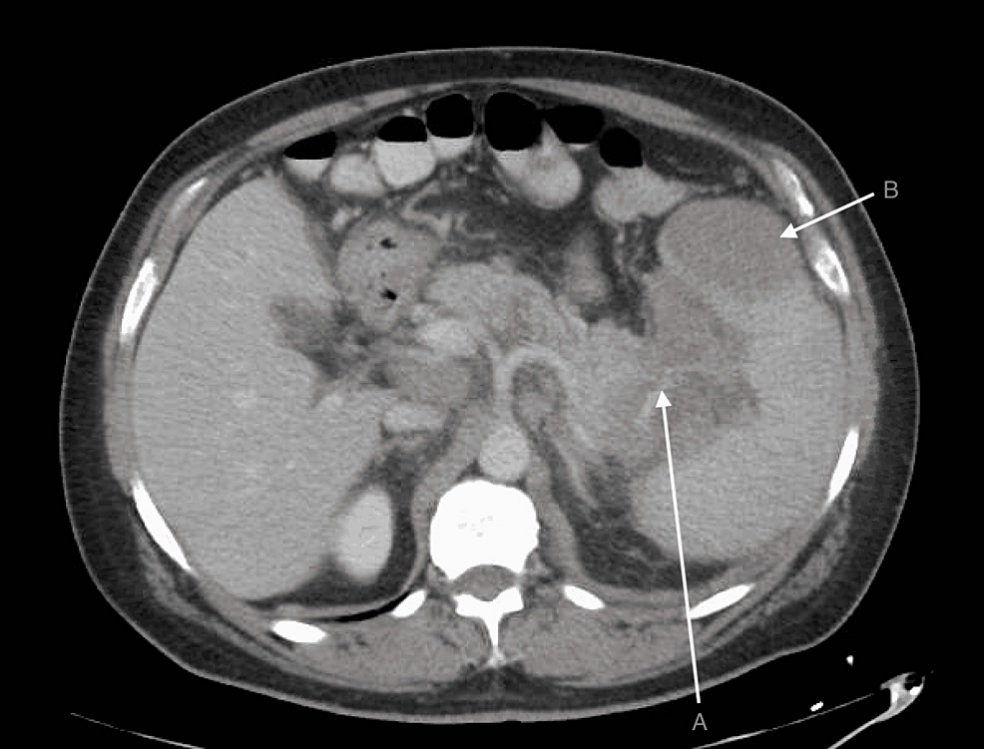
CT of the abdomen and pelvis demonstrating a large hypodense mass in the tail of the pancreas and a well-demarcated hypodense lesion in the anterior pole of the spleen A: Mass involving the tail of the pancreas extending and invading the splenic hilum. B: Mass within the anterior aspect of the spleen.

A CT scan of the abdomen and pelvis with intravenous contrast (Figure 1) showed a mass involving the tail of the pancreas invading the splenic hilum measuring approximately 6.1 cm x 8.7 cm in diameter. His spleen demonstrated mild to moderate splenomegaly with a well-defined 5.4 cm x 5.9 cm hypodense mass in the anterior pole. His CT also was notable for extensive lymphadenopathy involving the retroperitoneum, upper abdomen, and mesentery. An endoscopic ultrasound-guided fine needle aspiration (EUS-guided FNA) of the pancreatic mass was performed, which showed an abundant population of singly dispersed atypical lymphoid cells along with numerous lymphoglandular bodies in the background. His peripheral flow cytometry showed 16.49% abnormal cells with positive markers for CD10, CD19, CD20, and CD45 consistent with a diagnosis of B-cell lymphoma.

The patient was discharged from the hospital with a follow-up appointment with gastroenterology to discuss the cytology results of his EUS. He chose to receive treatment at a different hospital, so further details of his treatment plan were not available for discussion in this case report.

## Discussion

Diagnostic criteria for PPL have changed over the past 25 years; this case demonstrates how these evolving criteria suggest a rare presentation of PPL arising in the pancreatic tail without other constitutional symptoms.

Presenting symptoms of PPL are similar to those of other pancreatic malignancies. Patients may complain of vague nonspecific symptoms like dyspepsia, pain, nausea, vomiting, flatulence, an abdominal mass, and weight loss, typically with the absence of B symptoms like night sweats and fever [9]. Given the nonspecific nature of the presenting symptoms, characteristic imaging findings would be helpful in guiding a diagnosis. There have been studies that describe imaging findings suggestive of PPL over adenocarcinoma. Particularly, the presence of a bulky pancreatic head mass without necrosis or calcification, absence of significant pancreatic ductal dilatation, and diffuse pancreatic enlargement with invasive growth not respective of anatomical borders are more suggestive of PPL [10-12].

Despite the suggestive findings, clinical and radiographic presentation of PPL remain insufficient to confirm the diagnosis. Tissue histology, often obtained with minimally invasive procedures like percutaneous biopsy or EUS-guided FNA, is critical in appropriately diagnosing the patient. Accurate and early diagnosis allows prompt initiation of therapy, which differs between PPL and other common malignancies such as adenocarcinoma. In this case, EUS-guided FNA clearly diagnosed lymphoma but failed to delineate between primary and secondary types.

The significance of defining individuals with significant extra-pancreatic involvement, as demonstrated in this case, was likely of higher importance when surgical management of PPL was more common. As the role of chemotherapy for the management of PPL is now the mainstay of treatment in pancreatic lymphomas, it may be reasonable to adopt criteria for the diagnosis of PPL based on the predominantly diseased organ system, such as those proposed by the World Health Organization (WHO) [13]. In 2010, the WHO proposed a diagnosis of PPL if the bulk of the disease was confined to the pancreas despite the presence of adjacent lymphadenopathy or other metastatic diseases [14]. However, appropriately diagnosing lymphoma from other malignancies remains challenging without biopsy, and this case contributes to growing evidence of anatomical variation of PPL, which may help inform diagnosis.

## Conclusions

This case represents a rare localization of extranodal lymphoma to the pancreatic tail. Few other case reports exist with similar presentations, which is suggestive of a growing recognition of incidence in areas outside of the head.
